# Triple infection with pulmonary tuberculosis, chronic hepatitis C and systemic brucellosis in an HIV/AIDS patient: a case report from northwestern China

**DOI:** 10.3389/fmed.2026.1820404

**Published:** 2026-05-19

**Authors:** Xue Han, Quanlong Ma, Fang Wang, Xia Du, Zhongkun Liu, Xv Zhang

**Affiliations:** 1 Department of Infectious Disease, The Fourth People’s Hospital of Ningxia Hui Autonomous Region, Yinchuan, Ningxia, China; 2 Department of Infectious Disease, People’s Hospital of Tongxin County, Wuzhong, Ningxia, China; 3 Department of Infectious Disease, General Hospital of Ningxia Medical University, Yinchuan, Ningxia, China

**Keywords:** brucellosis, coinfection, hepatitis C virus, HIV/AIDS, tuberculosis

## Abstract

**Background and objectives:**

Profound immune suppression in people living with HIV/AIDS predisposes to multiple opportunistic and endemic infections. While HIV/tuberculosis (TB) and HIV/hepatitis C virus (HCV) coinfections are well recognized, simultaneous infection with *Mycobacterium tuberculosis*, HCV, and *Brucella* spp. is exceptionally rare. Such triple infection poses substantial diagnostic and therapeutic challenges, especially in endemic settings where overlapping febrile syndromes may obscure multiple concurrent etiologies. We describe a complex case of HIV/TB/HCV/brucellosis coinfection from northwestern China and summarize the diagnostic approach, staged treatment strategy, and long-term outcome.

**Case presentation:**

A 43-year-old man with a history of intravenous drug use and poorly controlled HIV infection presented with fatigue and progressive left scrotal swelling. He was found to have advanced HIV disease (CD4+ T-cell count 128/μL; HIV RNA 15,000 copies/mL), pulmonary TB confirmed by bronchoalveolar lavage fluid culture, chronic hepatitis C virus infection, and systemic brucellosis confirmed by blood culture and serology. Baseline liver biochemistry was within normal limits, although imaging suggested underlying chronic hepatic vulnerability. Initial treatment consisted of an individualized anti-tuberculosis regimen with isoniazid, rifampin, ethambutol, and levofloxacin, a 12-week rifampin–levofloxacin regimen for brucellosis, and ART with tenofovir disoproxil fumarate/lamivudine/efavirenz. HCV treatment was deferred because of concern about cumulative hepatotoxicity and contraindication between rifampin and direct-acting antivirals. Poor ART adherence subsequently led to virologic failure and resistance, prompting intensification to bictegravir/emtricitabine/tenofovir alafenamide. After completion of rifampin-based therapy, sofosbuvir/velpatasvir was initiated for HCV.

**Results:**

The patient’s course was marked by early treatment interruption, delayed virologic suppression, and later recovery under a simplified, multidisciplinary treatment strategy. At month 33, HIV RNA remained 83,500 copies/mL with a CD4+ T-cell count of 80/μL. By month 35, resistance-associated mutations had been detected and ART was intensified. At month 43, HIV RNA had declined to 79 copies/mL, allowing initiation of HCV treatment. By month 48, HIV RNA was 76 copies/mL, HCV RNA was < 25 IU/mL, and the CD4+ T-cell count had risen to 147/μL. At month 54, the patient had achieved substantial immune recovery (CD4+ T-cell count 520/μL) and durable HIV suppression (HIV RNA < 20 copies/mL), with no clinical or radiologic evidence of relapsed TB or brucellosis.

**Conclusion:**

This case highlights the diagnostic complexity of overlapping infectious syndromes in an immunocompromised host from an endemic region and demonstrates that even highly complex coinfections can be successfully managed through timely microbiologic confirmation, individualized sequencing of anti-infective and antiretroviral therapies, careful management of drug–drug interactions, and sustained adherence support.

## Introduction

Human immunodeficiency virus (HIV) remains a major global health challenge. According to the Joint United Nations Programme on HIV/AIDS (UNAIDS), an estimated 40.8 million people were living with HIV by the end of 2024 (1). As HIV infection progresses and cellular immunity declines, individuals become increasingly vulnerable to a wide spectrum of opportunistic infections. Tuberculosis (TB) is of particular concern and continues to be the leading cause of HIV-related mortality worldwide. A recent meta-analysis estimated that the global prevalence of HIV/TB coinfection is approximately 14% (2). In China, a systematic review reported a TB prevalence of 6.0% among people living with HIV/AIDS (PLWHA), lower than the previously reported 7.2%; however, when analyses were restricted to patients with AIDS, the prevalence rose to 22.8%, underscoring the heavy burden of HIV/TB coinfection in advanced disease (3, 4).

Because of shared routes of transmission, hepatitis C virus (HCV) infection is also common in people with HIV, particularly among individuals who inject drugs. Meta-analyses from the Asia-Pacific region, including large Chinese cohorts, have shown that the prevalence of HIV/HCV coinfection in China is around 24.7%–25.5%, while the prevalence of HCV coinfection reaches 81.6% in the subgroup of people who inject drugs (5, 6). HIV/HCV coinfection is associated with more rapid progression of liver fibrosis, an increased risk of cirrhosis and hepatocellular carcinoma, and a poorer response to antiretroviral therapy (ART), making its recognition and management a critical component of HIV care (7).

In endemic settings, however, the diagnostic challenge extends beyond any single coinfection. Acute or subacute febrile syndromes in resource-limited and livestock-exposed regions often have broad and overlapping etiologic spectra, including TB, viral infections, and under-recognized bacterial zoonoses (8, 9). Brucellosis in particular commonly presents as a non-specific febrile illness with constitutional symptoms, hepatosplenomegaly, and variable focal manifestations, and is therefore frequently misdiagnosed as other infectious causes of fever (9, 10). In immunocompromised patients, this overlap may be even more pronounced, making early etiologic clarification particularly difficult when multiple concurrent infections are present.

Brucellosis, caused by *Brucella* spp., is a zoonotic bacterial infection that remains endemic in many parts of the world, including northwestern China. Human infection typically results from direct contact with infected livestock or consumption of unpasteurized dairy products. Although brucellosis is well recognized in the general population of endemic regions, it continues to be reported only rarely as an opportunistic infection in people with HIV/AIDS. A recent review from Mediterranean countries found no strong overall association between HIV infection and brucellosis, yet data from specific settings suggest that coinfection can be substantial: in one Iranian cohort, brucellosis accounted for 26.3% of bacterial infections among hospitalized HIV-positive patients, and a study from Africa reported a brucellosis seroprevalence of 2.48% in HIV-infected pregnant women (11–13). In China, only sporadic case reports of HIV–brucellosis coinfection have been published to date, but in regions where zoonotic diseases are endemic, the potential impact of brucellosis on immunocompromised PLWHA deserves close attention (14).

To date, reports of triple infection with *Mycobacterium tuberculosis*, HCV and *Brucella* spp. in a single HIV/AIDS patient are exceedingly rare. Overlapping and non-specific clinical manifestations, the risk of significant drug–drug interactions and cumulative hepatotoxicity, and atypical presentations related to immune suppression all contribute to substantial diagnostic and therapeutic complexity in such cases.

Here, we describe a rare and diagnostically challenging case of a 43-year-old man with HIV/AIDS living in a brucellosis-endemic area of northwestern China, who was simultaneously infected with pulmonary TB, chronic HCV and systemic brucellosis. This case highlights the importance of comprehensive pathogen screening, a heightened index of suspicion for multiple coinfections in immunocompromised hosts, and close multidisciplinary collaboration in the management of complex clinical scenarios. By presenting this unique case, we aim to add to the limited literature on HIV/AIDS patients harboring multiple endemic and opportunistic infections and to underline the diagnostic and therapeutic challenges posed by such triple infections.

## Case presentation

A 43-year-old man residing in Ningxia, northwestern China, presented to our hospital in March 2021 with a 2-month history of fatigue and progressive swelling of the left hemiscrotum. He reported a history of intravenous drug use and had been diagnosed with HIV infection in 2011 through routine screening. However, he had deferred initiation of ART until 2020 and admitted to poor adherence thereafter. There was no documented history of tuberculosis, viral hepatitis, or brucellosis, although he reported frequent contact with livestock—particularly sheep—in his rural hometown.

On admission, his vital signs were stable, with a temperature of 37.8 °C, blood pressure of 116/72 mmHg, heart rate of 82 beats/min, and respiratory rate of 18 breaths/min. Physical examination revealed marked enlargement and tenderness of the left hemiscrotum (approximately 3 cm × 5 cm), together with hepatosplenomegaly. No superficial lymphadenopathy was detected, and pulmonary auscultation was unremarkable.

Given his advanced immunocompromised state, history of injecting drug use, livestock exposure, and systemic symptoms, a broad and stepwise diagnostic work-up was initiated on admission. Baseline laboratory testing showed a CD4+ T-cell count of 128/μL and an HIV RNA load of 15,000 copies/mL, consistent with advanced HIV disease. Baseline liver biochemistry, including alanine aminotransferase, aspartate aminotransferase, and total bilirubin, was within normal limits. Interferon-γ release assay was positive (276.1 pg/mL), and chest computed tomography (CT) demonstrated a cavitary lesion in the left upper lobe suggestive of post-primary pulmonary TB. Subsequent *Mycobacterium tuberculosis* drug resistance gene detection (XpretMTD/RIF) detected *Mycobacterium tuberculosis* and rifampicin sensitivity.

In parallel, screening for blood-borne viral infection revealed positive anti-HCV serology and an HCV RNA level of 1.07 × 10^7^ IU/mL, confirming chronic HCV infection. Abdominal ultrasonography demonstrated coarse hepatic echotexture and hepatosplenomegaly, while transient elastography showed a liver stiffness of 7.4 kPa and a controlled attenuation parameter of 202 dB/m, indicating no significant hepatic steatosis but suggesting underlying chronic hepatic vulnerability despite preserved baseline biochemical liver function.

Because the patient lived in a brucellosis-endemic area and reported frequent livestock exposure, systemic brucellosis was also suspected. Blood cultures yielded *Brucella* spp., and the standard tube agglutination test (SAT) titre was 1:400. Together with his compatible clinical manifestations—fatigue, low fever, hepatosplenomegaly, and painful scrotal swelling—and an elevated erythrocyte sedimentation rate (41 mm/h), these findings established the diagnosis of systemic brucellosis. Taken together, the patient was diagnosed with WHO stage IV HIV/AIDS complicated by pulmonary TB, chronic HCV infection, and systemic brucellosis, representing a rare and clinically complex triple infection in an immunocompromised host from a brucellosis-endemic region.

**FIGURE 1:**
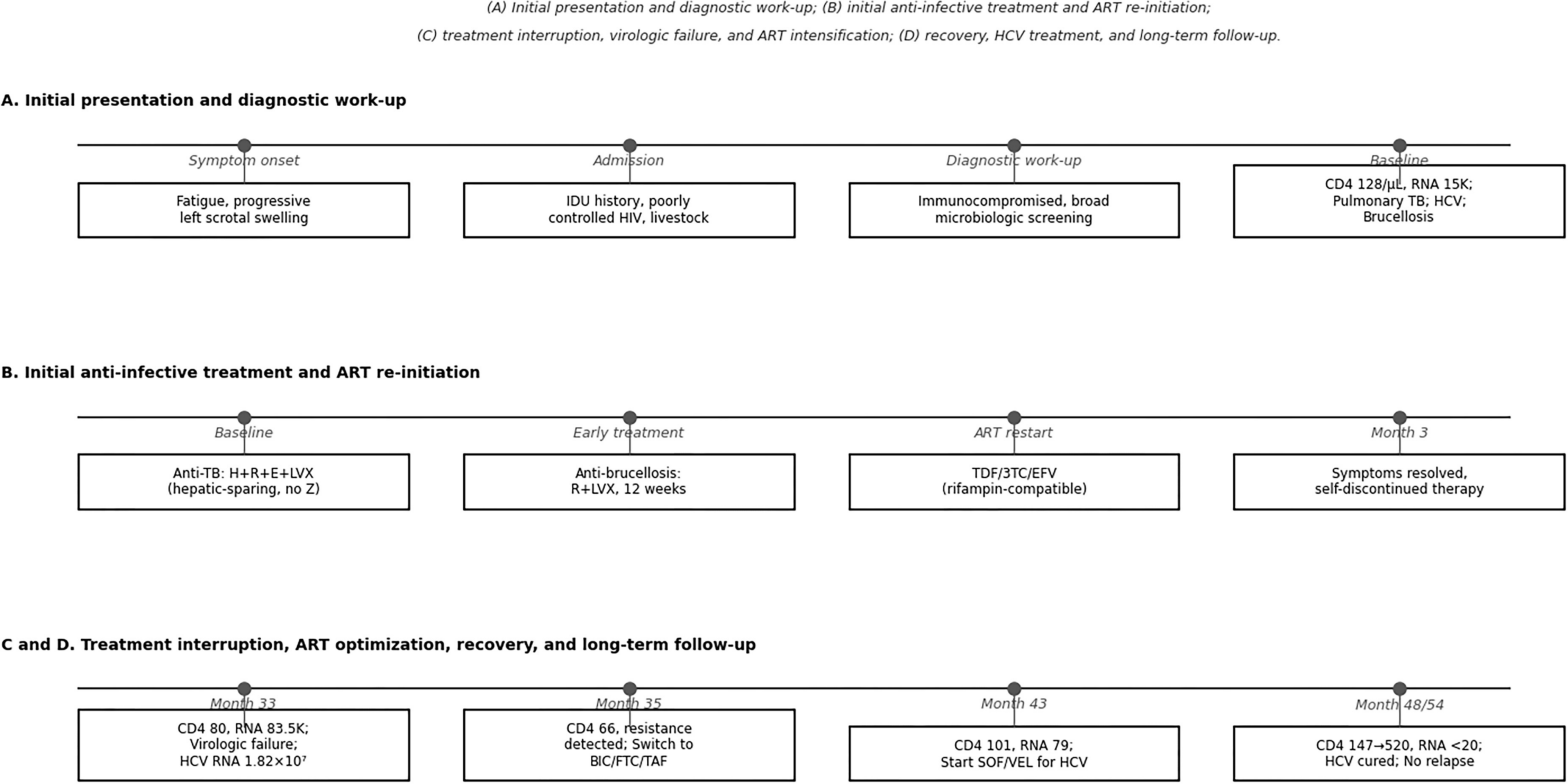
Timeline of clinical presentation, diagnostic milestones, therapeutic interventions, and long-term follow-up in a patient with HIV/AIDS, pulmonary tuberculosis, chronic hepatitis C, and systemic brucellosis. The figure summarizes symptom onset, hospital admission, confirmation of advanced HIV disease, microbiologic diagnosis of pulmonary tuberculosis and systemic brucellosis, confirmation of chronic HCV infection, initiation of anti-tuberculosis and anti-brucellosis therapy, re-initiation and later intensification of antiretroviral therapy, delayed initiation of direct-acting antiviral treatment for HCV, and major follow-up milestones over 54 months. **(A)** Initial presentation and diagnostic work-up; **(B)** initial anti-infective treatment and ART re-initiation; **(C)** treatment interruption, virologic failure, and ART intensification; **(D)** recovery, HCV treatment, and long-term follow-up.

A chronological summary of the major clinical events, diagnostic findings, treatment milestones, and follow-up assessments is provided in Table 1, and the overall disease trajectory is illustrated in Figure 1.

**TABLE 1:** Chronological summary of key clinical events, investigations, therapeutic interventions, and follow-up outcomes.

Time point	Clinical event	Main clinical features	Key investigation results	Main interventions
March 2021 (day 0)	Initial presentation and admission.	Fatigue; progressive left scrotal pain and swelling; hepatosplenomegaly.	Left hemiscrotum markedly enlarged and tender (∼3 cm × 5 cm).	Hospital admission
Day 1	Initial HIV assessment.	Low fever (37.8°C); systemic symptoms persisted.	CD4+ T-cell count 128/μL; HIV RNA 15,000 copies/mL.	ART reinitiated with TDF/3TC/EFV
Day 3	Initial diagnosis of pulmonary TB and systemic brucellosis.	Symptoms persisted.	IGRA positive (276.1 pg/mL); SAT 1:400; chest CT showed a cavitary lesion in the left upper lobe; abdominal ultrasound showed coarse hepatic echotexture, splenomegaly, splenic vein dilatation, and a splenic hilar hypoechoic nodule; scrotal ultrasound showed bilateral epididymo-orchitis.	Anti-TB therapy initiated with INH + RIF + EMB + LFX
Day 5	Confirmation of chronic HCV infection.	Symptoms persisted.	HCV RNA positive (final source-confirmed value to be unified throughout manuscript).	HCV treatment deferred
Day 6	Microbiologic confirmation of TB.	Normothermia, the rest symptoms persisted.	*Mycobacterium tuberculosis* drug resistance gene detection (XpertMTD/RIF) detected *Mycobacterium tuberculosis* and rifampicin sensitivity.	Continued current regimen
Day 10	Early treatment response.	Scrotal pain improved; fatigue lessened.	Complete blood count and liver/renal function remained within normal limits.	Continued treatment
Day 11	Discharge.	Clinically improved.	–	Discharged with anti-TB, anti-brucellosis, and ART plan
Month 3	Telephone follow-up.	Scrotal pain/swelling resolved; fatigue resolved.	No formal laboratory reassessment available.	Patient reported completion of anti-brucellosis therapy and partial completion of anti-TB therapy
December 2023 (month 33)	Outpatient follow-up.	No major symptoms.	CD4+ T-cell count 80/μL; HIV RNA 83,500 copies/mL; HCV RNA remained high; SAT negative; complete blood count and liver/renal function normal; chest CT showed no new abnormalities.	No regimen change at this visit
February 2024 (month 35)	Virologic failure and resistance evaluation.	No major new symptoms; ART adherence poor.	CD4+ T-cell count 66/μL; HIV genotypic resistance testing suggested reduced susceptibility to zidovudine and tenofovir; abdominal ultrasound showed coarse hepatic echotexture and splenomegaly (11.1 cm × 4.2 cm); SAT negative; chest CT stable.	ART intensified to BIC/FTC/TAF
November 2024 (month 43)	Follow-up after ART intensification.	Clinically stable.	CD4+ T-cell count 101/μL; HIV RNA 79 copies/mL; transient elastography: liver stiffness 11.5 kPa, CAP 208 dB/m; complete blood count and liver/renal function normal; chest CT showed no new lesions.	SOF/VEL initiated for HCV; continued BIC/FTC/TAF
March 2025 (month 48)	Intermediate long-term follow-up.	Clinical improvement maintained.	CD4+ T-cell count 147/μL; HIV RNA 76 copies/mL; HCV RNA < 25 IU/mL; complete blood count and liver/renal function normal; abdominal ultrasound showed persistent mild splenomegaly (12.4 cm × 4.1 cm).	Continued BIC/FTC/TAF
September 2025 (month 54)	Final follow-up.	Improved energy; stable weight.	CD4+ T-cell count 520/μL; HIV RNA < 20 copies/mL; complete blood count and liver/renal function normal; no evidence of active TB or relapsed brucellosis.	Continued follow-up on optimized ART

ART, antiretroviral therapy; BALF, bronchoalveolar lavage fluid; BIC/FTC/TAF, bictegravir/emtricitabine/tenofovir alafenamide; CAP, controlled attenuation parameter; EFV, efavirenz; EMB, ethambutol; HCV, hepatitis C virus; IGRA, interferon-γ release assay; INH, isoniazid; LFX, levofloxacin; RIF, rifampin; SAT, standard tube agglutination test; SOF/VEL, sofosbuvir/velpatasvir; TB, tuberculosis; TDF/3TC/EFV, tenofovir disoproxil fumarate/lamivudine/efavirenz. Xpret MTB/RIF, drug resistance gene detection for *Mycobacterium tuberculosis*.

## Diagnostic assessment

The diagnostic assessment was guided by the need to distinguish multiple potentially overlapping infectious etiologies in a severely immunocompromised patient from an endemic region. With respect to HIV disease status, baseline testing at admission showed a CD4+ T-cell count of 128/μL and an HIV RNA load of 15,000 copies/mL, consistent with advanced HIV infection and WHO clinical stage IV disease. This degree of immunodeficiency substantially increased the risk of opportunistic and regionally endemic infections and justified a broad microbiologic evaluation.

TB was strongly suspected because of the patient’s persistent fatigue, low fever, night sweats, systemic symptoms, and residence in a high-burden setting. Interferon-γ release assay was positive, and chest CT demonstrated a cavitary lesion in the left upper lobe compatible with post-primary pulmonary TB. Subsequent *Mycobacterium tuberculosis* drug resistance gene detection (XpretMTD/RIF) detected *Mycobacterium tuberculosis* and rifampicin sensitivity.

Given the history of injecting drug use, viral hepatitis screening was also performed. Anti-HCV antibody was positive and HCV RNA was 1.07 × 10^7^ IU/mL, confirming chronic HCV infection. Although baseline liver biochemistry was not overtly abnormal, abdominal ultrasonography showed coarse hepatic echotexture and hepatosplenomegaly, suggesting chronic liver involvement relevant to subsequent treatment planning.

Systemic brucellosis was considered because of frequent livestock exposure in a brucellosis-endemic area. Blood cultures yielded *Brucella* spp., and the SAT titre was 1:400. The patient also had non-specific but compatible manifestations, including fatigue, low fever, hepatosplenomegaly, and painful scrotal swelling, together with an elevated erythrocyte sedimentation rate. These findings supported the diagnosis of active systemic brucellosis.

Several differential diagnoses were considered and systematically excluded. In the setting of advanced immunosuppression, non-tuberculous mycobacterial infection could not initially be ruled out; however, BALF culture specifically yielded *M. tuberculosis*, with no evidence of non-tuberculous mycobacteria. HIV-related lymphoma was also considered because of constitutional symptoms and splenomegaly, but the absence of lymphadenopathy on physical examination and imaging, together with microbiologic confirmation of multiple infectious pathogens, made a primary hematologic malignancy less likely. Other bacterial systemic infections, including *Salmonella* and *Leptospira* spp., were considered, but the exposure history and microbiologic findings did not support these alternatives. Autoimmune or granulomatous disorders such as sarcoidosis were considered less likely given the microbiologic confirmation of both TB and brucellosis.

Overall, the final diagnosis of advanced HIV/AIDS with concurrent pulmonary TB, chronic HCV infection, and systemic brucellosis was established through integration of the clinical presentation, imaging findings, serology, and microbiologic confirmation.

## Therapeutic interventions

In view of the patient’s advanced immunosuppression, high HIV RNA load, and confirmed coinfection with pulmonary TB, chronic HCV, and systemic brucellosis, a multidisciplinary, staged treatment strategy was adopted. The principal objectives were: (1) to control active infections; (2) to minimize drug–drug interactions and cumulative toxicity; and (3) to optimize long-term adherence to ART.

Initial anti-TB therapy consisted of isoniazid, rifampin, ethambutol, and levofloxacin. Pyrazinamide was intentionally omitted because of concern for cumulative hepatotoxicity in the setting of untreated chronic HCV infection and imaging evidence of chronic liver involvement, despite normal baseline liver biochemistry. Levofloxacin was included both to maintain a four-drug anti-mycobacterial regimen after omission of pyrazinamide and to provide overlapping activity against *Brucella* spp. during the initial treatment phase. We recognize that this was an individualized, liver-sparing strategy rather than a standard first-line regimen for uncomplicated drug-susceptible pulmonary TB.

Brucellosis was treated with rifampin plus levofloxacin for 12 weeks. This combination was selected pragmatically to align with the rifampin-containing TB regimen, reduce pill burden, and avoid adding doxycycline-related gastrointestinal intolerance in a patient already facing a complex multidrug schedule. Alternative doxycycline-based regimens were considered, but were deferred in favor of regimen simplicity and therapeutic overlap during the acute phase.

Initial ART consisted of tenofovir disoproxil fumarate (TDF), lamivudine (3TC), and efavirenz (EFV). This regimen was chosen as a pragmatic, individualized option at the time of ART re-initiation in 2021. EFV was selected primarily because of its relative compatibility with rifampin-based therapy, which was required concurrently for pulmonary TB and brucellosis. In addition, the patient had a history of delayed ART initiation, poor prior adherence, and social vulnerability related to injecting drug use and unstable treatment engagement; therefore, a relatively simple once-daily regimen with broad local availability was considered more feasible at that stage than more complex alternatives. Baseline genotypic resistance testing had not been performed at the time of ART re-initiation, and resistance was identified only later, after virologic failure became evident during follow-up.

HCV treatment was intentionally deferred during the initial phase of TB and brucellosis management. This decision reflected both concern about cumulative hepatotoxicity and the known contraindication between rifampin and sofosbuvir/velpatasvir. However, the eventual initiation of SOF/VEL was also delayed well beyond completion of rifampin-based therapy because of the patient’s financial constraints.

Taken together, these treatment decisions reflected a staged, compromise-based strategy designed to balance microbiologic efficacy, hepatic safety, drug–drug interaction management, and regimen feasibility in an unusually complex coinfected host.

## Follow-up and outcomes

At month 3 follow-up, conducted by telephone, the patient reported that scrotal pain and swelling had resolved and fatigue had disappeared. He stated that he had completed anti-brucellosis therapy and approximately three months of anti-tuberculosis treatment, but had discontinued both regimens prematurely after symptomatic improvement. He continued ART without formal supervision. Because this follow-up was conducted remotely, no sputum examination, chest imaging, or in-person clinical reassessment could be performed at that time. Shortly thereafter, the patient left his hometown for migrant work and was unavailable for regular outpatient follow-up, resulting in a prolonged gap in tuberculosis-specific monitoring after treatment interruption.

At the month 33 follow-up visit (December 2023), laboratory testing showed a CD4+ T-cell count of 80/μL and persistent HIV viremia of 83,500 copies/mL, indicating inadequate immune recovery and virologic failure. HCV RNA remained high at 1.82 × 10^7^ IU/mL. *Brucella* serology was negative, complete blood count and liver and renal function tests were within normal limits, and repeat chest CT showed no new abnormalities. Sputum smear examination performed at that visit was also negative. Although there was no radiologic evidence of progressive TB and no apparent relapse of brucellosis, his immunovirologic status remained concerning.

By the month 35 visit (February 2024), ART adherence had further deteriorated, partly because of nausea and vomiting, and the CD4+ T-cell count had declined to 66/μL. HIV genotypic resistance testing, which was performed after virologic failure became evident, identified mutations associated with reduced susceptibility to zidovudine and tenofovir. Abdominal ultrasonography showed coarse hepatic echotexture and persistent splenomegaly (11.1 cm × 4.2 cm). *Brucella* serology remained negative, and chest imaging continued to show no clinically significant change. In response to virologic failure and emerging resistance, ART was intensified to a once-daily, single-tablet integrase strand transfer inhibitor–based regimen consisting of bictegravir/emtricitabine/tenofovir alafenamide. At the same time, a multidisciplinary team involving infectious diseases physicians, case managers, and social support staff implemented structured adherence counseling and close follow-up.

At approximately month 43 (November 2024), laboratory evaluation showed partial immunologic recovery, with a CD4+ T-cell count of 101/μL, and effective virologic suppression, with HIV RNA reduced to 79 copies/mL. Chest CT showed no new lesions or evidence of active TB. Transient elastography demonstrated a liver stiffness of 11.5 kPa and a controlled attenuation parameter of 208 dB/m, while abdominal ultrasonography showed persistent mild splenomegaly. In the setting of improved HIV control and completion of rifampin-based therapy, pan-genotypic HCV treatment with sofosbuvir/velpatasvir was initiated. Importantly, SOF/VEL was started approximately 40 months after rifampin had been discontinued, so no specific washout interval needed to be considered at that stage. The prolonged delay in HCV treatment initiation was mainly related to the patient’s financial constraints rather than persistent concern about residual rifampin enzyme induction.

At the month 48 follow-up (March 2025), the patient showed further clinical and virologic improvement. The CD4+ T-cell count had risen to 147/μL, HIV RNA was 76 copies/mL, and HCV RNA had declined to < 25 IU/mL, indicating effective suppression of both HIV and HCV. Complete blood counts and liver enzyme levels remained within normal ranges. Abdominal ultrasonography continued to show mild splenomegaly without evidence of hepatic decompensation.

By the month 54 follow-up visit (September 2025), the patient had achieved substantial and sustained immunovirologic recovery. The CD4+ T-cell count had increased to 520/μL, and HIV RNA was < 20 copies/mL. Hematologic indices and liver and renal function remained normal, chest imaging showed no evidence of active TB, and there were no clinical or laboratory signs of relapsed brucellosis. The patient reported improved energy levels and stable weight. Overall, despite early challenges related to poor adherence, treatment interruption, delayed viral suppression, and later detection of resistance, the implementation of a patient-centered, multidisciplinary care strategy resulted in a favorable long-term outcome.

## Discussion

This case illustrates a rare and diagnostically challenging scenario of triple infection in a patient with HIV/AIDS, involving pulmonary TB, chronic HCV infection, and systemic brucellosis. Each of these infections is clinically significant on its own; however, their coexistence in a single immunocompromised host markedly complicates diagnosis, therapeutic decision-making, and long-term management. To our knowledge, there are very few reports worldwide of individuals living with HIV/AIDS who are simultaneously infected with *M. tuberculosis*, HCV, and *Brucella* spp. The present case is therefore clinically relevant not only because of the rarity of this pathogen combination, but also because it demonstrates how infections with different transmission routes, disease kinetics, and treatment constraints may converge in one vulnerable host.

Because of impaired cell-mediated immunity, patients with advanced HIV infection are at heightened risk for both opportunistic and regionally endemic infections. TB remains the leading cause of AIDS-related death globally, and HIV infection increases the risk of developing active TB by approximately 16–27-fold compared with the general population (15). In the present case, the diagnosis of TB was supported by characteristic chest CT findings and microbiologic confirmation of *M. tuberculosis* in BALF culture. HCV coinfection is also common among people with HIV, particularly among those with a history of injecting drug use, as in this patient. HIV/HCV coinfection is known to accelerate the progression of liver fibrosis, increase the risk of cirrhosis and hepatocellular carcinoma, and may attenuate the response to ART (7). In our patient, the diagnosis of chronic HCV was established early, but its management had to be carefully integrated into a broader therapeutic sequence shaped by TB and brucellosis treatment requirements.

An important feature of this case is that it can also be understood within the framework of febrile syndromes in endemic regions. In settings where TB and zoonotic infections coexist, non-specific systemic manifestations such as fatigue, low-grade fever, weight loss, sweats, and hepatosplenomegaly may reflect more than one active pathogen at the same time (8, 9). This is particularly relevant in immunocompromised patients, in whom symptom expression may be blunted, atypical, or misleading. Brucellosis is especially prone to delayed recognition because it often presents with constitutional symptoms and variable focal organ involvement that overlap with other infectious syndromes (9, 10). In our patient, fatigue, splenomegaly, and scrotal swelling could initially have been attributed to HIV-related systemic illness, TB, or other bacterial infections. The case therefore highlights the need for broad microbiologic thinking rather than single-pathogen anchoring when evaluating complex febrile presentations in endemic settings.

Brucellosis is a zoonotic bacterial disease that remains endemic in northwestern China. Although it is only rarely reported among people living with HIV, coinfection is plausible in endemic areas where livestock exposure is common (11–14). In the present case, systemic brucellosis was confirmed by positive blood cultures for *Brucella* spp. and a diagnostic SAT titre of 1:400, on a background of compatible clinical features. This underscores the importance of including brucellosis in the differential diagnosis of systemic febrile illness in immunocompromised patients residing in endemic regions (14). It also reinforces that exposure history remains highly informative even in the era of broad molecular and microbiologic diagnostics, particularly for zoonotic infections that may otherwise be overlooked.

The simultaneous treatment of TB, HCV, brucellosis, and HIV presents substantial therapeutic challenges because of overlapping toxicities and complex drug–drug interactions. Rifampin is central to the management of both TB and brucellosis, but it is a potent inducer of hepatic cytochrome P450 enzymes and drug transporters, it can significantly reduce serum concentrations of many antiretroviral agents (16). The initial choice of TDF/3TC/EFV should therefore be understood in its clinical context. At the time of treatment re-initiation, the patient required rifampin-based therapy, had a history of poor adherence and delayed ART engagement, and did not yet have baseline resistance data available. Under these circumstances, EFV-based ART represented a practical compromise between rifampin compatibility, regimen simplicity, local accessibility and the need to maximize the likelihood of adherence. The later switch to BIC/FTC/TAF was made only after treatment interruption, virologic failure and resistance testing had demonstrated that a more potent and better-tolerated regimen was required. Later, once TB therapy had been completed and virologic failure with resistance had emerged, ART was escalated to an INSTI-based single-tablet regimen (BIC/FTC/TAF). This strategy avoided clinically significant interactions with direct-acting antiviral (DAA) therapy and allowed durable HIV viral suppression without compromising HCV treatment efficacy.

The rationale for omitting pyrazinamide and including levofloxacin deserves particular emphasis. Although the patient’s baseline ALT, AST, and bilirubin were not elevated, he had untreated chronic HCV infection together with coarse hepatic echotexture and hepatosplenomegaly on imaging, making cumulative hepatotoxicity a major concern. Because pyrazinamide is widely regarded as the most hepatotoxic component of standard first-line TB therapy, a more liver-sparing approach was adopted at treatment initiation (17). In patients with chronic liver disease or increased hepatic vulnerability, individualized anti-tuberculosis regimens that limit the number of hepatotoxic agents and incorporate a fluoroquinolone as a companion drug have been recommended as a reasonable alternative strategy (18). Levofloxacin was therefore selected as an additional relatively non-hepatotoxic companion drug and, at the same time, provided overlapping antibacterial activity against *Brucella* spp., since quinolones have documented *in vitro* and clinical efficacy in human brucellosis and may be considered in combination regimens (19). More recent comparative evidence also suggests that fluoroquinolone-containing combinations may provide acceptable clinical efficacy in selected brucellosis settings, although they are not universally preferred over conventional doxycycline-based regimens (20). This case-specific strategy was thus designed to balance microbiologic coverage, hepatic safety, and regimen feasibility in a patient requiring simultaneous treatment of TB, brucellosis, and HIV.

Several alternative management strategies merit explicit consideration. First, for drug-susceptible pulmonary TB, the conventional regimen would have been HRZE. However, in this patient, the coexistence of untreated chronic HCV infection, imaging evidence of chronic liver involvement, and the need for concurrent rifampin-based treatment for brucellosis made cumulative hepatotoxicity a dominant concern. Second, alternative ART/TB cotreatment strategies could have included rifabutin-based TB therapy to facilitate a protease inhibitor– or INSTI-based ART backbone, or dolutegravir-based ART with dose adjustment during rifampin coadministration (16). In the present case, however, TDF/3TC/EFV represented the most practical initial choice because of rifampin compatibility, local accessibility, and the need for a relatively simple regimen in a patient with delayed ART initiation and poor adherence. Third, with respect to brucellosis, a doxycycline-based regimen would have been more conventional and may have stronger support in the literature, but was not chosen because of the need to reduce pill burden and gastrointestinal intolerance while maintaining overlap with rifampin-based therapy. Finally, earlier initiation of HCV treatment was theoretically possible, but sofosbuvir/velpatasvir was deferred because coadministration with rifampin is contraindicated due to marked reduction in antiviral exposure and risk of treatment failure (21). In the present case, however, SOF/VEL was not initiated shortly after rifampin discontinuation. Instead, treatment began approximately 40 months after rifampin had been stopped, which was far beyond any clinically relevant residual enzyme-inducing effect. Accordingly, no formal pharmacologic washout period was specifically considered when SOF/VEL was eventually started. The main reason for this prolonged delay was financial limitation rather than ongoing concern about rifampin-related drug interaction. This distinction is important for clinical interpretation: although interaction-driven postponement was appropriate during rifampin-based therapy, the much later initiation of direct-acting antiviral treatment in this case reflected a real-world socioeconomic barrier rather than a pharmacologic necessity.

These considerations indicate that the final regimen used in this case should not be viewed as a universal template, but rather as one individualized pathway selected in the context of competing priorities.

A further clinically important issue in this case was the patient’s premature discontinuation of anti-tuberculosis therapy after approximately three months. This interruption was particularly concerning because the baseline chest CT showed a cavitary lesion and the initial regimen did not include pyrazinamide, making the effective treatment duration even more limited than in standard short-course therapy. From a clinical perspective, such non-adherence could have increased the risk of persistent bacillary burden, relapse, and treatment failure. However, after the month 3 telephone follow-up, the patient left for migrant work and was lost to regular in-person tuberculosis follow-up, so no immediate sputum or imaging reassessment could be performed. When he returned for reassessment at month 33, chest CT and sputum smear examination did not show evidence of active pulmonary tuberculosis. Although this does not completely exclude the possibility of subclinical persistence during the interval, it suggests that no overt microbiologic or radiologic relapse had become apparent by the time of re-evaluation. This episode highlights the practical difficulties of managing tuberculosis in socially mobile and medically vulnerable patients, and it underscores the importance of continuity of follow-up, adherence support, and renewed imaging and microbiologic reassessment whenever treatment interruption occurs.

The patient’s inconsistent adherence to ART contributed to delayed viral suppression and the emergence of resistance mutations, a common and critical barrier to HIV control in socially vulnerable populations such as people who inject drugs. Structured adherence support—including case management, simplified once-daily single-tablet ART, and regular counseling—played a pivotal role in ultimately achieving durable immunovirologic recovery. By Months 43, 48, and 54, the patient’s CD4+ T-cell count had progressively improved, HIV RNA remained in the low-copy range or undetectable, and HCV viral load had been successfully suppressed. By the final follow-up, there was no clinical or radiologic evidence of TB or brucellosis relapse. This favorable long-term outcome suggests that even when initial management is compromised by non-adherence and delayed virologic control, substantial recovery remains possible if treatment can later be simplified, reinforced, and sustained.

This case also carries broader public health implications. First, it underscores the need for comprehensive infectious disease screening in people living with HIV/AIDS who reside in, or originate from, endemic regions where multiple pathogens such as *M. tuberculosis*, HCV, and *Brucella* spp. may co-circulate. Second, it illustrates the importance of rational treatment sequencing, enabling clinicians to balance efficacy and safety in the face of overlapping toxicities and major pharmacokinetic interactions. Third, it highlights the continuing importance of sustained adherence support not only for HIV treatment success and resistance prevention, but also for maintaining continuity of anti-tuberculosis therapy and reducing the risk associated with premature treatment interruption, particularly in high-risk and socially mobile groups such as people who inject drugs. Finally, the favorable outcome in this case underscores the value of a multidisciplinary, patient-centered approach integrating infectious diseases specialists, pulmonologists, hepatologists, pharmacists, and adherence counselors to deliver coordinated care. In endemic settings, clinicians should remain alert not only to single coinfections, but also to the possibility of multiple concurrent infectious etiologies requiring flexible, staged, and individualized management.

## Conclusion

This case highlights the clinical complexity of triple infection with pulmonary tuberculosis, chronic HCV infection, and systemic brucellosis in a patient with advanced HIV/AIDS from an endemic region. Its main significance lies not only in the rarity of this pathogen combination, but also in the diagnostic challenge created by overlapping febrile and systemic manifestations in an immunocompromised host.

Our patient’s favorable long-term outcome illustrates that even highly complex coinfections can be successfully managed through timely microbiologic confirmation, individualized sequencing of anti-infective and antiretroviral therapies, careful attention to drug–drug interactions, and sustained adherence support. This report therefore emphasizes the need for clinicians in endemic settings to maintain a broad differential diagnosis and to adopt flexible, patient-centered strategies when multiple chronic and opportunistic infections coexist.

## Data Availability

The raw data supporting the conclusions of this article will be made available by the authors, without undue reservation.
